# Multiple Wheat Genomes Reveal Novel *Gli-2* Sublocus Location and Variation of Celiac Disease Epitopes in Duplicated α-Gliadin Genes

**DOI:** 10.3389/fpls.2021.715985

**Published:** 2021-09-03

**Authors:** Gwyneth Halstead-Nussloch, Tsuyoshi Tanaka, Dario Copetti, Timothy Paape, Fuminori Kobayashi, Masaomi Hatakeyama, Hiroyuki Kanamori, Jianzhong Wu, Martin Mascher, Kanako Kawaura, Kentaro K. Shimizu, Hirokazu Handa

**Affiliations:** ^1^Department of Evolutionary Biology and Environmental Studies, University of Zurich, Zurich, Switzerland; ^2^Institute of Crop Science, National Agriculture and Food Research Organization, Tsukuba, Japan; ^3^Research Center for Advanced Analysis, National Agriculture and Food Research Organization, Tsukuba, Japan; ^4^Molecular Plant Breeding, Institute of Agricultural Sciences, ETH Zürich, Zurich, Switzerland; ^5^Brookhaven National Laboratory, Upton, NY, United States; ^6^Functional Genomics Center Zurich, Zurich, Switzerland; ^7^Leibniz Institute of Plant Genetics and Crop Plant Research (IPK), Gatersleben, Germany; ^8^German Centre for Integrative Biodiversity Research (iDiv) Halle-Jena-Leipzig, Leipzig, Germany; ^9^Kihara Institute for Biological Research, Yokohama City University, Yokohama, Japan; ^10^Graduate School of Life and Environmental Sciences, Kyoto Prefectural University, Kyoto, Japan

**Keywords:** α-gliadin, celiac disease epitopes, copy number variation, *Gli-2* loci, wheat (*Triticum aestivum* L.)

## Abstract

The seed protein α-gliadin is a major component of wheat flour and causes gluten-related diseases. However, due to the complexity of this multigene family with a genome structure composed of dozens of copies derived from tandem and genome duplications, little was known about the variation between accessions, and thus little effort has been made to explicitly target α-gliadin for bread wheat breeding. Here, we analyzed genomic variation in α-gliadins across 11 recently published chromosome-scale assemblies of hexaploid wheat, with validation using long-read data. We unexpectedly found that the *Gli-B2* locus is not a single contiguous locus but is composed of two subloci, suggesting the possibility of recombination between the two during breeding. We confirmed that the number of immunogenic epitopes among 11 accessions varied. The D subgenome of a European spelt line also contained epitopes, in agreement with its hybridization history. Evolutionary analysis identified amino acid sites under diversifying selection, suggesting their functional importance. The analysis opens the way for improved grain quality and safety through wheat breeding.

## Introduction

Since its origin by allopolyploidization, bread wheat (*Triticum aestivum* L.) has become a staple crop, providing ∼20% of the calories consumed globally (Shiferaw et al., 2013). Concentrated breeding efforts have increased yield such that the production of bread wheat reached 766 million tons in 2019 (FAOSTAT, 2021). Further selection has made wheat more palatable and increased the quality of desired end-use traits.

Wheat grains are typically processed into flour to make various breads and noodles. Much of the rheological quality of these products relies on gluten formation. Gluten is a complex of two protein families, glutenins and gliadins, which are storage proteins in wheat endosperm. Gliadins are classified into three groups, α-, γ-, and ω-gliadins, based on their electrophoretic mobility (Shewry and Halford, 2002). α-Gliadins are the most abundant gliadins and represent 15–30% of the wheat seed protein (Gu et al., 2004). Genes encoding α-gliadins are tandemly duplicated and form clusters within each *Gli-2* locus. They are located on the short arm of homoeologous chromosome group 6 in *Triticum* species (Qi et al., 2013; Ozuna et al., 2015). Because *T. aestivum* is allohexaploid (AABBDD), it contains three *Gli-2* loci called *Gli-A2*, *Gli-B2*, and *Gli-D2* (Payne, 1987). High allelic diversity, copy number variation, and expression differences in α-gliadins in bread wheat have been attributed to the combination of tandem and whole genome duplications (Salentijn et al., 2009; Noma et al., 2016; Huo et al., 2018). Although allelic diversity, gene copy number variation and other differences in α-gliadins may be linked to the phenotypic differences for the wheat flour qualities among the cultivars, little is known about precise genomic information for the *Gli-2* loci to provide a basis for comparison between cultivars. For example, the general function of α-gliadins in the breadmaking quality is well understood, but the role of individual α-gliadin genes is not entirely clear (Branlard et al., 2001; Brennan, 2009; Metakovsky et al., 2018). α-Gliadins are also the most common proteins that trigger an immune response in patients with celiac disease (CD), one of the widespread wheat-related health disorders (Scherf et al., 2016). The CD reaction is caused by the presence of a variety of peptide sequences called epitopes (Sollid et al., 2012; Juhász et al., 2018). The repetitive domain includes the DQ2.5-glia-α1, DQ2.5-glia-α2, and DQ2.5-glia-α3 epitopes (Ozuna et al., 2015), and sometimes, these epitopes overlap to create a 33-mer peptide that is highly immunotoxic to celiac patients (Shan et al., 2002; Huo et al., 2018; Juhász et al., 2018). The immunotoxicity of the 33-mer region was verified by genome-editing (Sánchez-León et al., 2018). Although the three-dimensional structure of a short CD epitope bound to human HLA has been reported (Kim et al., 2004; Petersen et al., 2014, 2016), little is known about the higher-order structure of gliadin proteins because they aggregate in solutions (Urade et al., 2018). Sequence-based characterization of α-gliadin variation within modern hexaploid wheat cultivars will aid in breeding efforts to incorporate both desired end-use quality and lower reactivity for consumers.

Allopolyploidization and tandem duplication have made regions such as *Gli-2* difficult to characterize in terms of the genomic organization of and variation within multigene families found in bread wheat. Thus, most variation within α-gliadin gene sequences of different wheat accessions and related species has been detected using bacterial artificial chromosome (BAC) clones, transcriptome analysis, or low-coverage shotgun genome sequencing (Kawaura et al., 2012; Noma et al., 2016; Juhász et al., 2018). High resolution of the structure of homoeologous *Gli-2* loci has been described using long-read sequences, but in only one cultivar, Chinese Spring (CS; Huo et al., 2018). Recently, advances in polyploid genomics enabled the high-quality genome assembly and polymorphism analysis of tandem duplications (Paape et al., 2016, 2018; Avni et al., 2017). Here, using chromosome-level assemblies for 11 accessions including elite bread wheat cultivars and a spelt wheat line in the framework of the “10+ Wheat Genomes Project” (Walkowiak et al., 2020), we began to address the question of global variation in both the structure of and polymorphism within *Gli-2* loci among multiple cultivars.

## Materials and Methods

### Sequence Resources

Reference-quality genome assemblies for 9 bread wheat accessions, Arina*LrFor*, CDC Landmark, CDC Stanley, Jagger, Julius, LongReach Lancer, Mace, Norin 61, SY Mattis and one spelt accession, PI190962, released by the “10+ Wheat Genomes Project” (Walkowiak et al., 2020), were accessed through IPK, Germany.^1^ We also used the RefSeq v1.0 assembly of CS (International Wheat Genome Sequencing Consortium (IWGSC) et al., 2018), which is available at INRAE, France.^2^

### Identification of *Gli-2* Loci and α-Gliadin Sequences

To identify the location of the *Gli-2* loci, BLAST searches were conducted against chromosome assemblies for homoeologous group 6 of the eleven accessions using the α-gliadin gene sequences AS2 and AS7 (for chromosome 6A); AS3, AS4, AS5 and AS6 (for 6B); and AS1, AS8, AS9, AS10, and AS11 (for 6D) as queries (Noma et al., 2016). From the BLAST results, regions with an *e*-value = 0 and composed of a single exon were selected as candidates for α-gliadin gene copies. The regions were translated into amino acid sequences. Sequences not starting with a methionine residue were discarded as incomplete gene fragments. Sequences that were too diverged based on the sequence alignment or phylogenetic tree were also omitted. Finally, we constructed a codon-based alignment of gliadin gene copies using MUSCLE in MEGA (Kumar et al., 2018). Hi-C data and the alignments of CDC Landmark Oxford Nanopore Technologies (ONT) long-read data were obtained from Walkowiak et al. (2020). The gene coverage values of the long-read alignments were obtained with SAMtools v1.0 (Li et al., 2009) and BEDtools v2.29.0 (Quinlan and Hall, 2010). Read alignments were visualized with IGV v2.8.2 (Robinson et al., 2017).

The evolutionary history of the gene family was inferred from the 429 α-gliadin sequences identified above. Codon positions included were 1st + 2nd + 3rd + Non-coding. All positions containing gaps and missing data were eliminated (complete deletion option). The final dataset contained a total of 524 positions. The tree was estimated using the neighbor-joining method (Saitou and Nei, 1987), and evolutionary distances were computed using the Kimura 2-parameter method (Kimura, 1980) and expressed as the number of base substitutions per site. The rate variation among sites was modeled with a gamma distribution (shape parameter = 2.25). Support for the tree topology was estimated using the bootstrap test with 1,000 replicates and was calculated as the percentage of replicate trees in which the associated taxa clustered together (Felsenstein, 1985). The tree was drawn to scale, with the units for branch lengths being the same as those of the evolutionary distances used to infer the phylogenetic tree.

### Celiac Disease Epitope Search and Site Selection Analysis

Using the amino acid sequences of α-gliadin copies without the last stop codon, we searched all sequences for the presence of nine canonical amino acid epitopes previously shown to induce an immunogenic reaction (Sollid et al., 2012; Ozuna et al., 2015).

To test for amino acid sites likely to be under positive selection in the α-gliadin gene family, only full-length sequences were considered for a conservative analysis. Gaps present at the same position in all three *Gli-2* loci and sequences containing premature stop codons were discarded. Sequences were also removed if they had no terminal stop codon or were not composed of multiples of three nucleotides, implying frameshifts. Last, sites in regions that were difficult to align (the polyglutamine regions) were not considered, as the uncertain alignments may produce false positive signals. For the selection analysis, a phylogenetic method was applied. First, the most likely phylogenetic tree was estimated using nucleotide alignment and a general time reversible (GTR) + invariant + gamma model in MrBayes (Ronquist et al., 2012). Then, the likelihood of that tree was calculated under different codon substitution models by estimating the non-synonymous and synonymous substitution rate ratios (ω = *d*_N_/*d*_S_) for each codon within the alignment. The value of ω indicates the type of selection: ω < 1 indicates negative selection, ω = 0 indicates neutral evolution, and ω > 1 indicates positive selection. A likelihood ratio test (LRT) was run between two nested codon substitution models, a null and an alternative model, to determine whether the alternative model of positive selection was supported. The null model (M7) did not allow for sites under positive selection while the alternative model (M8) did allow for positive selection (Yang et al., 2000). Last, the posterior probability of a specific site being under positive selection was estimated using Bayesian empirical Bayes (BEB) (Yang et al., 2005). Sites with a probability > 95% were considered significant. The likelihoods of the codon substitution models and posterior probability calculations were implemented in the CODEML program of the software package PAML4 (Yang, 2007).

## Results

### Location and Validation of *Gli-2* Loci in Accessions

We identified α-gliadin gene copies within 11 wheat assemblies: the 10 reference-quality pseudomolecule assemblies (Walkowiak et al., 2020) plus CS RefSeq v1.0 (International Wheat Genome Sequencing Consortium (IWGSC) et al., 2018). We first examined the chromosomal positions of the α-gliadin copies. Copies that mapped to chromosome 6A in the 11 wheat accessions were assigned as *Gli-A2* and were located in single region on the short arm, as expected (Table 1 and Supplementary Table 1). The only exception was *Gli-A2* of CDC Landmark, which was split into 2 subloci 7 Mb apart from each other. Similarly, sequences in *Gli-D2* mapped to the expected region on chromosome 6D in nine reference-quality assemblies. In LongReach Lancer and CS, we could not identify α-gliadin copies on chromosome 6D; however, those found in scaffolds that were not anchored to a chromosome (chrUn) were assigned to *Gli-D2* following the suggestion of Juhász et al. (2018). Surprisingly, the copies found on chromosome 6B showed that the *Gli-B2* locus was clearly split into 2 subloci in all accessions. We called them *Gli-B2-1* and *Gli-B2-2*, and they were 12–21 Mb apart from each other on chromosome 6B (Table 1 and Supplementary Table 1). The uniformity of the Hi-C signal along the whole *Gli-B2* region and its flanking regions further supported that the bipartite structure of *Gli-B2* was not an assembly artifact (Supplementary Figure 1) (Shimizu et al., 2020; Walkowiak et al., 2020). The position of this second locus relative to the well-described locus at ∼43 Mb on chromosome 6B in CS has not been described before, although previous studies mention two sequences that mapped outside that region (Huo et al., 2018; Juhász et al., 2018). The consistency in the Hi-C maps observed among all assemblies supports that *Gli-B2* is composed of two parts and opens the possibility of exploiting genetic recombination for breeding purposes.

**TABLE 1:** Genomic positions of *Gli-2* loci in 11 wheat accessions.

**Accession**	***Gli-A2***		***Gli-B2***						***Gli-D2***	
	Positions*	Copy no.**	Gli-B2-1*	Copy no.**	Gli-B2-2*	Copy no.**	Gli-B2-3*	Copy no.**	Positions*	Copy no.**
Arina*LrFor*	25.5–27.7	30 (14)	44.5–45.1	6 (4)	63.0	2 (1)			28.1–28.5	9 (5)
CDC Landmark	25.8–26.4 and 33.0–33.1	11 (9)	43.4–43.6 and 49.5–49.7	12 (6)	63.2	2 (1)			27.1–27.5	9 (5)
CDC Stanley	26.1–27.0	11 (9)	45.3–46.0	12 (7)	65.2	2 (1)			28.2–28.7	10 (6)
Jagger	24.7–25.6	11 (9)	43.6–44.4	9 (3)	65.0	2 (1)	654.9–655.2	6 (4)	26.7–26.8	3 (1)
Julius	24.7–25.5	10 (8)	46.6–47.1	6 (3)	64.7	2 (0)			27.0–27.4	9 (5)
LongReach Lancer	24.6–25.4	11 (9)	35.5–35.7 and 45.7–46.0	14 (6)	58.2	2 (1)			chrUn	11 (8)
Mace	24.8–25.6	11 (9)	43.5–44.3	13 (7)	63.8	2 (1)			26.8–27.3	11 (7)
Norin 61	25.8–28.2	33 (15)	42.3–43.0	14 (8)	61.2	2 (1)			26.7–27.2	11 (6)
SY Mattis	24.0–25.9	23 (8)	44.6–45.2	6 (2)	61.8	2 (0)			26.6–27.1	9 (5)
PI190962 (spelt)	25.8–28.2	25 (10)	41.3–41.7	5 (2)	60.4	2 (1)			26.4–26.9	11 (6)
Chinese Spring	24.9–25.6	9 (7)	43.4–44.1	8 (3)	62.7	2 (1)			chrUn	18 (12)

Genomic positions (in Mb) and copy number of α-gliadin genes that mapped within chromosome 6A, 6B, or 6D of each accession were assigned to *Gli-A2*, *Gli-B2*, and *Gli-D2*, respectively. Within LongReach Lancer and Chinese Spring, copies that mapped to chrUn were assigned to *Gli-D2*. *Genomic positions (Mb) in the pseudomolecules of the corresponding chromosomes. **The number of intact genes encoded in this region is shown in parentheses.

Although some assemblies showed the subdivision or translocation of several α-gliadin genes compared to those of other accessions, we interpreted them with caution. In Jagger, α-gliadin sequences mapped to a third region (*Gli-B2-3*) located at the end of the long arm of chromosome 6B. We also found that the sublocus *Gli-B2-1* of LongReach Lancer and CDC Landmark was further split into two parts. However, the Hi-C signal of intra- and interchromosomal interactions for these accessions suggested potential misassembly within these regions (Supplementary Figures 1, 2). We note that the 20 Mb regions flanking *Gli*-B2-1 in CDC Landmark were highly concordant with those in CS, but they were not concordant with those in LongReach Lancer (Supplementary Figure 3). Because the assembly structure and orientation of CS was also supported by additional evidence (International Wheat Genome Sequencing Consortium (IWGSC) et al., 2018), this suggested that the rearrangement in CDC Landmark may represent true biological variation. The location and orientation of these subloci remain interesting cases for further validation to distinguish biological rearrangement from assembly errors.

Next, we checked the accuracy of the assemblies around each single α-gliadin gene copy. We utilized the long-read sequence data from ONT for CDC Landmark that was previously used to validate the assembly (Walkowiak et al., 2020). Though often flanked by assembly gaps, the sequence at and immediately adjacent to each α-gliadin gene copy was supported by continuous alignments of several long reads (Supplementary Figure 4), implying a gene-level correctness of each model. The different coverage seen among copies, including those in close proximity, hinted at potential collapses of paralogous copies into a single gene (Supplementary Figure 4B) or the separate assembly of allelic heterozygous copies. To address this possibility, we compared the coverage of the ONT alignments for each α-gliadin gene in the assembly to the median genome-wide gene coverage (32.23 genome equivalents). Of the 34 copies that we manually annotated in CDC Landmark, seven (20.5%) had a mean coverage that clearly deviated from that of other copies. As a comparison, the coverage of the three *ADH* homoeologs (chosen as a single-copy gene reference) was well within the genome-wide value (Supplementary Table 1). While the three α-gliadin copies at high coverage likely represent collapsed paralogs, the four genes at lower coverage may be haplotype-specific assemblies of heterozygous allelic copies. The long-read data suggested that the assembled α-gliadin sequences were correctly identified, although the exact copy number of ∼20% of them may be different.

The number of assembled α-gliadin genes within each *Gli-2* locus is reported in Table 1. While most accessions possessed approximately 11 α-gliadin copies in *Gli-A2*, the accessions Arina*LrFor*, Norin 61, SY Mattis, and PI190962 had two to three times as many copies (Table 1). We identified 13–17 copies in *Gli-B2* in most accessions, while Arina*LrFor*, Julius, SY Mattis and the European spelt PI190962 had only half the number of copies compared to the other accessions (Table 1). For *Gli-D2*, there were approximately 10 copies in most accessions (Table 1). An extremely high or low copy number for *Gli-B2* and *Gli-D2* in Jagger, respectively, was possibly an assembly error, as described above. Subsequent analyses in this paper will use the assignment to a particular *Gli-2* locus based on previously published assemblies for consistency.

### Phylogenetic Analysis of α-Gliadin Copies

**FIGURE 1:**
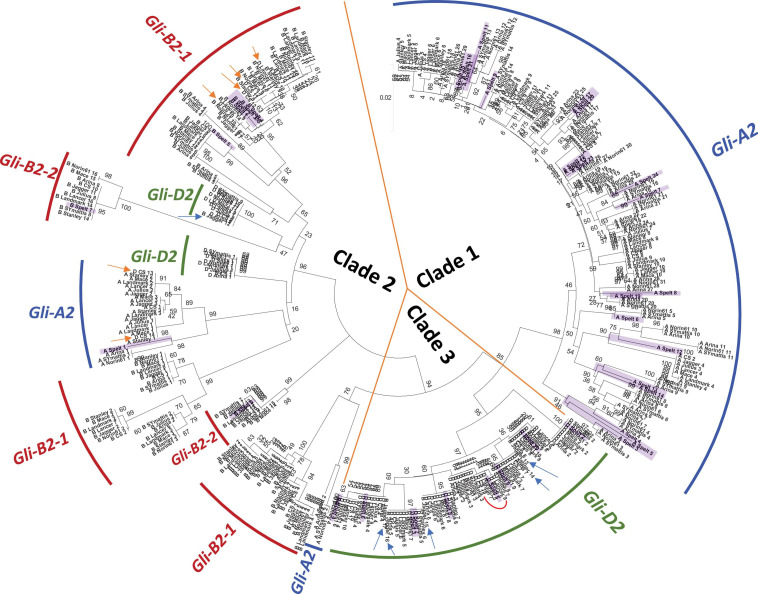
Phylogenetic relationship of α-gliadin copies in 11 wheat accessions, including spelt. The 429 α-gliadin copies showed clustering based on subgenome assignment. The evolutionary tree is largely divided into three clades. Each subclade is indicated by the arcs and colored according to the corresponding *Gli-2* loci. Numbers at branch splits are bootstrap percentages. Copies assigned to loci to which they do not cluster are indicated by arrows: *Gli-B2-3* in Jagger are in blue, and *Gli-D2* in CS and LongReach Lancer are in orange. Spelt copies are highlighted in light purple. The red curve in clade 3 highlights the seven sequences containing the immunotoxic 33-mer.

We then assessed the relationship between all α-gliadin copies identified in the 11 accessions using phylogenetic analysis. According to the clustering pattern, α-gliadin copies were classified into three main clades named 1, 2, and 3 (Figure 1). Clades 1 and 3 showed a compact structure and included copies mostly from *Gli-A2* to *Gli-D2*, respectively. In *Gli-D2*, unlike other subgenome loci, there was little difference in copy number between accessions and the genetic distances between branches were shorter. Limited allelic diversity at the *Gli-D2* locus is consistent with the lower diversity of the coding sequences in the D subgenome (International Wheat Genome Sequencing Consortium (IWGSC), 2014; Jordan et al., 2015; Walkowiak et al., 2020). Clade 2 mostly contained copies from *Gli-B2* but also included sub-clades of *Gli-A2* and *Gli-D2*, although with weak branch support (Figure 1). As mentioned above, we found 2 subloci in *Gli-B2*, i.e., *Gli-B2-1* and *Gli-B2-2* (Table 1). In the phylogenetic tree (Figure 1), the two gliadin sequences encoded in *Gli-B2-2* formed subclades distinct from other sequences in *Gli-B2-1*, indicating that the split of *Gli-B2* was shared among all wheat accessions and that the genes in the two subloci experienced different histories. These data further support the bipartite structure of *Gli-B2*.

The α-gliadin copies that mapped to Jagger *Gli-B2-3* clustered in clade 3, which is composed of *Gli-D2* copies (Figure 1, blue arrows). This is consistent with the possible misassembly from *Gli-D2* to the end of chromosome 6BL in this cultivar (see also the previous section; Supplementary Figure 2). If we reassign these sequences as a part of *Gli-D2*, the copy numbers of *Gli-B2* and *Gli-D2* in Jagger are closer to the average copy number found within the other accessions. In the case of LongReach Lancer and CS, we assigned all α-gliadin copies in chrUn as copies of *Gli-D2* following the suggestion of Juhász et al. (2018). However, we found that several copies clustered with those assigned to *Gli-A2* or *Gli-B2* in the other accessions (Figure 1, orange arrows). Despite these potential misclassifications, we were able to show that there were clear variations among accessions.

Among the accessions with the largest differences in copy numbers, we observed distinct clustering patterns. Branches from accessions with the highest copy number for *Gli-A2*, such as Arina*LrFor*, Norin 61, SY Mattis and PI190962, were clearly separated from the branches of the other seven accessions. Similarly, we found distinct clusters containing copies of *Gli-B2-1* from Arina*LrFor*, Julius, and SY Mattis. These three accessions, in addition to PI190962, contained the lowest copy number within this locus. These examples highlight potential differences in evolutionary and/or breeding history between accessions and that the gene duplications or losses in some cultivars did not originate independently but were likely from a common ancestor.

Focusing on the spelt wheat, PI190962, we observed no clear association with the other accessions for *Gli-A2* and *Gli-B2-1*. Rather, most of the copies in PI190962 formed their own branches or small clusters. This was not the case for *Gli-D2*, where the PI190962 copies were positioned on the same branches as those for other bread wheats. Interestingly, the α-gliadin copies in *Gli-B2-2* in PI190962 also clustered with those of the other accessions (Figure 1, purple highlight). The spelt accession, PI190962, used in this study is a Central European spelt, which has been suggested to have originated from the introgression of a hulled tetraploid emmer wheat into bread wheat during the migration of bread wheat from the Fertile Crescent to Europe. Therefore, the A and B subgenomes between bread wheat and European spelt had higher sequence divergence, while the D subgenome showed greater sequence similarity (Blatter et al., 2004; Dvorak et al., 2012). Our observation of the separation of α-gliadin sequences in PI190962 from those of other bread wheats in *Gli-A2* and *Gli-B2-1* supports that this Central European spelt accession had an introgressed origin with a tetraploid emmer wheat, which was recently shown to be distinct from the origins of Iberian spelt (Abrouk et al., 2021). This result also indicates that the introgressed loci from chromosome 6B of emmer wheat may be confined to the region encoding *Gli-B2-1*, further supporting a different evolutionary history for the two *Gli-B2* loci identified in this study.

### Celiac Disease Epitope Copy Number and Positive Selection in α-Gliadins

**FIGURE 2:**
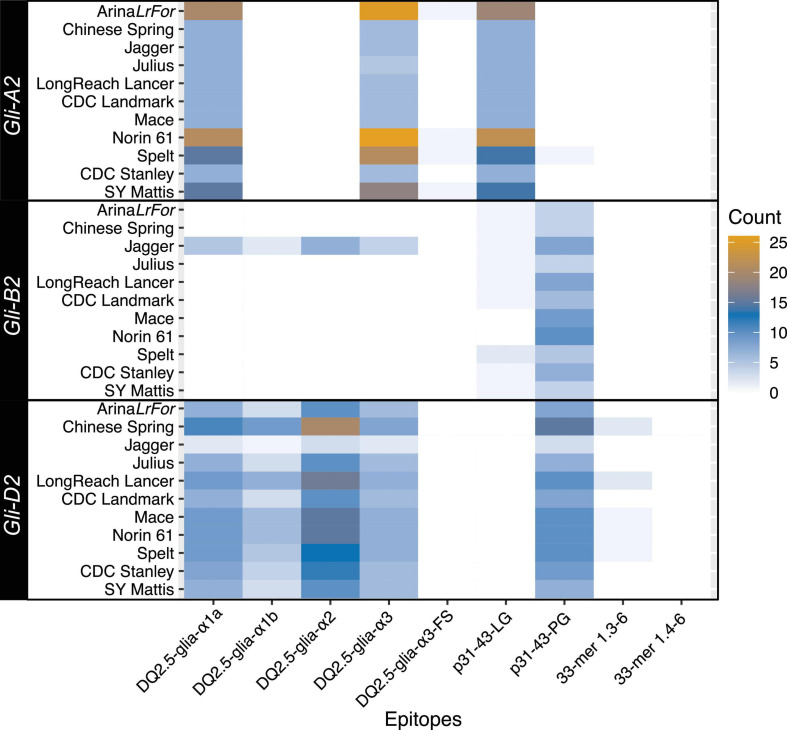
Celiac disease (CD) epitope quantification within α-gliadin copies. The frequency of canonical CD epitopes varies between accessions and homoeologous chromosomes.

Specific epitopes found in α-gliadins can induce reactions in patients with CD and gluten intolerance. Therefore, the search for new alleles and/or copy number variations that may cause weaker or no reaction is beneficial in breeding programs. Among the amino acid sequences produced by α-gliadin genes from the 11 wheat accessions, we found polymorphic sites within three major immunogenic regions, p31-43, the 33-mer, and the DQ2.5-glia-α3 peptide, using the established nomenclature (Sollid et al., 2012; Ozuna et al., 2015). The presence of epitope sequences showed a subgenome-specific pattern within the 11 accessions (Figure 2), and the count of CD epitopes in each accession mirrored the total α-gliadin copy number present in each locus (Table 1). *Gli-A2* contained mostly DQ2.5-glia-α1b, DQ2.5-glia-α3 and p31-43-LG epitopes. Variants of the latter two epitopes were also present, but at low frequency, in four accessions. The B subgenome encoded the fewest epitopes, the highest proportion of which were p31-43-PG. Among all accessions, the largest variety of CD epitopes was present in *Gli-D2* and included several that overlapped in a single gene copy. The toxic 33-mer sequence that contains six epitopes (33-mer 1.3-6) was found in the *Gli-D2* sequence of 5 accessions, including once in PI190962 and twice each in CS and LongReach Lancer (Figure 1, red curve and Figure 2).

In general, a single known epitope sequence was not found in the genes of all three subgenomes and sequences with multiple overlapping epitopes were restricted to *Gli-D2*. For example, DQ2.5glia-α1a was present in both *Gli-A2* and *Gli-D2*. The sequence encoding both DQ2.5glia-α1a and DQ2.5glia-α2 (PFPQPQLPYPQ) was found only in *Gli-D2* due to a P to S substitution (PFPQPQLPYSQ) in *Gli-A2*. No DQ2.5-glia-α-type epitopes were present in *Gli-B2*, except for the potential misassembly or translocation of regions from chromosomes 6D to 6B in Jagger. The patterns we observed reflected those of previous studies reporting the presence of specific epitopes in the subgenomes of hexaploid wheat (van Herpen et al., 2006; Salentijn et al., 2009; Sollid et al., 2012; Ozuna et al., 2015; Noma et al., 2016; Juhász et al., 2018).

Global prevalence of CD has increased (Singh et al., 2018) and this has been attributed, by some, to modern breeding practices. Due to its hybridization history, spelt wheats contain different gliadin and glutenin contents and has been subject to less intensive selection than modern bread wheats (Dubois et al., 2016; Escarnot et al., 2018), prompting the idea it could be less reactive for consumers. We observed that the numbers and distribution patterns of the immunogenic epitopes in the particular spelt accession, PI190962, were similar to those of other bread wheat accessions (Figure 2), including one copy of the 33-mer peptide that was identified in *Gli-D2*. Although the study of Asian and other spelts (Blatter et al., 2004; Dvorak et al., 2012) would be necessary to draw conclusions about spelt diversity, the data from this single accession of spelt did not support the claim that spelt (as a species) could produce weaker reactions in people with CD, in agreement with previous genetic studies (Ozuna et al., 2015; Dubois et al., 2016; Ruiz-Carnicer et al., 2019). Recent studies investigating overall protein and gluten content of both modern and old hexaploid wheat as well as “ancient” varieties including spelt, emmer, and einkorn showed no conclusive role of modern breeding techniques in the increased prevalence of CD. Rather, they exemplified the high variability of gluten content between all varieties, new and old, and reiterate the importance of environmental factors in overall protein content of wheat and its relatives (Escarnot et al., 2018; Geisslitz et al., 2019; Call et al., 2020; Pronin et al., 2021). Our results on the genetic variability are in line with these protein-based studies and, taken together, show the tools to identify low immunoreactive varieties are well developed. These studies not only proposed suitable varieties for further breeding already but also motivate a more comprehensive characterization of wheat and its relatives to tap into existing variability for breeding (Shewry, 2018).

**FIGURE 3:**
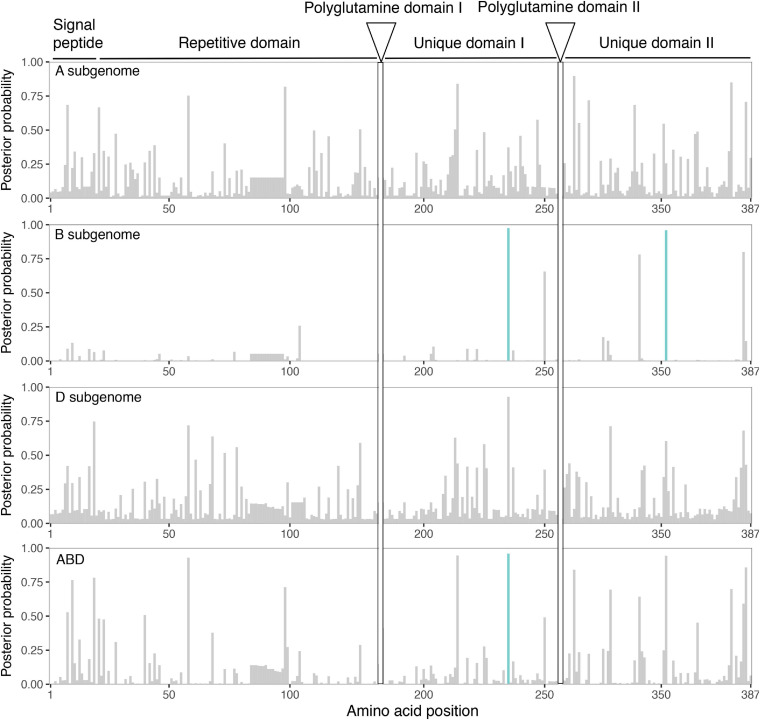
Sites under positive selection in α-gliadin. Amino acids under selection were detected by estimating the ratio of non-synonymous (dN) to synonymous substitutions (dS) in each codon in the α-gliadin alignment for each subgenome. The panels show the Bayesian posterior probability of an amino acid site being under positive selection for the gliadin genes of each subgenome separately and the alignment of all three together (ABD). Highlighted in blue are the sites with a posterior probability > 95%. The different conserved domains (from Noma et al., 2016) are marked at the top of the panels. Sites that were difficult to align in the polyglutamine domains are excluded.

We used a method to identify selection on amino acid-changing substitutions (PAML; Yang, 2007). This method estimates the ratio of amino-acid replacement mutations (non-synonymous substitutions, dN) compared with synonymous substitutions (dS). When the dN:dS ratio is greater than 1, it indicates positive or diversifying selection. Many positions showed a posterior probability higher than 0.75. Among them, in *Gli-B2*, we found two codon positions that were above the 95% significance level: one in unique domain I and another in unique domain II (Figure 3). When all *Gli-2* loci were analyzed together, the position in unique domain I remained significant (Figure 3 and Supplementary Table 2). The other amino acid position that was significant in *Gli-B2* domain II was just below the threshold when A, B and D were analyzed together (Figure 3).

## Discussion

The importance of bread wheat in human nutrition and its role in disease warrant the characterization of genetic and structural variation within the gene family encoding gliadin, which forms the gluten protein structure together with glutenin. However, this research has been challenging due to the complexity of the loci caused by tandem and homoeologous duplications. Here, we characterized the diversity of α-gliadin gene copies and their organization within *Gli-2* loci in chromosome-scale assemblies of 11 globally distributed bread and spelt wheat accessions. Long-read data supported that the assembled gliadin coding regions were correct, and 80% of them were assembled as a single copy with high confidence. The remaining 20% may possibly be collapsed, highly similar paralogs or independently assembled alleles of a gene copy. Unexpectedly, we found a bipartite structure of the *Gli-B2* loci in all assemblies, which was supported by Hi-C data and evolutionarily supported by phylogenetic analysis. This suggests that further expansion of the variation at the gliadin locus through chromosomal recombination using the segregation of these subloci may be applicable for future wheat breeding. Using the PAML method, we detected amino acid positions that were under diversifying selection, suggesting that polymorphisms at these positions may be relevant for functional differences, such as those involved in interactions with glutenins (Li et al., 2014). This warrants further functional validation via amino acid substitution experiments.

Previous reports describe the subgenome specificity of sequences with CD epitopes, and those that cause the strongest immune cell reactions occur mostly in the A and D subgenomes and their respective progenitors. On the other hand, the wheat B subgenome, barley and several other Triticeae species contain epitopes that produce relatively weak responses from their α-gliadin and related proteins (van Herpen et al., 2006; Juhász et al., 2018). Our results not only reflect this subgenome specificity but also show that epitopes causing gluten-related reactions are unevenly distributed among accessions covering a wide range of wheat diversity (Walkowiak et al., 2020). The D subgenome is the only identified source of the toxic 33-mer epitope within bread wheat, and its presence has been detected at low frequency in the germplasm of the D progenitor *Aegilops tauschii* (Schaart et al., 2021). Current efforts to incorporate this knowledge into breeding safer varieties include the generation of synthetics and *Gli-D2* deletion lines (Camerlengo et al., 2017; Li et al., 2018), the development of probes to quickly confirm the presence of reactive epitopes (Dubois et al., 2017), and the genome-editing to reduce the immunotoxic 33-mer (Sánchez-León et al., 2018). Our study can inform these efforts. Our results show the reduced frequency of reactive epitopes in some accessions but also show that reactive epitopes are present in spelt, which is consistent with a previous study (Escarnot et al., 2018), indicating that detailed cultivar-specific analysis is needed. While the immunogenic effects of many of the polymorphic epitopes have not been directly tested, our main findings indicate that resources for breeding less reactive wheat are already present in elite germplasm.

## Data Availability Statement

Publicly available datasets were analyzed in this study. This data can be found here: IPK, Germany (https://wheat.ipk-gatersleben.de/) INRAE, France (https://wheat-urgi.versailles.inra.fr/Seq-Repository/Assemblies).

## Author Contributions

KKS and HH conceived the study. TT, FK, HK, JW, and KK performed the identifications and phylogenetic analyses of α-gliadin genes. GH-N, DC, TP, and MH performed the epitope identification and evolutionary analyses within α-gliadins. DC and MM performed and curated the bioinformatic data. KK, KKS, and HH acquired the funding. GH-N, DC, KKS, and HH wrote the original draft. All authors contributed to the article and approved the submitted version.

## Conflict of Interest

The authors declare that the research was conducted in the absence of any commercial or financial relationships that could be construed as a potential conflict of interest.

## Publisher’s Note

All claims expressed in this article are solely those of the authors and do not necessarily represent those of their affiliated organizations, or those of the publisher, the editors and the reviewers. Any product that may be evaluated in this article, or claim that may be made by its manufacturer, is not guaranteed or endorsed by the publisher.

## Footnotes

1
https://wheat.ipk-gatersleben.de/

2
https://wheat-urgi.versailles.inra.fr/Seq-Repository/Assemblies

## Supplementary Material

Supplementary Figure 1 Large-scale chromosome organization around the *Gli-B2* loci in 11 accessions. Hi-C contact matrices of chromosome 6B at 0–100 Mb show a strong signal on the diagonal and a relatively even gradient perpendicular to the diagonal, indicating that the large-scale structure of assemblies is correct. Dashed lines represent the position of each sub locus identified in *Gli-B2*.

Supplementary Figure 2 Interchromosomal interactions between chromosomes 6B and 6D for each accession. Interactions between these chromosomes are weak apart from small regions that show dark vertical lines (e.g., in Jagger, Landmark, Mace), representing evidence of possible misassembly.

Supplementary Figure 3 Alignment of LongReach Lancer and CDC Landmark chromosome 6B to that of Chinese Spring (CS). In the region from 0 to 100 Mb on 6B, LongReach Lancer may have inversions compared with CS. On the other hand, though interrupted by assembly gaps and indels, the region is assembled in CDC Landmark with the same orientation as that in CS.

Supplementary Figure 4 Validation of the *Gli-2* loci with long-read data. Oxford Nanopore Technologies long reads of CDC Landmark were aligned to the short read-based assembly of the same variety (Walkowiak et al., 2020). Alignments at the *Gli-2* loci were inspected for overall structure [**(A)**, *Gli-A2a* on chr6A 25.7–26.5 Mb] and at the single-gene scale [**(B)** *Gli-B2-1a* on chr6B]. Despite the presence of assembly gaps, the sequence of the coding regions was well supported by multiple reads.

Supplementary Table 1 Gene copy name, chromosome with start and stop position, gene annotation (if available) from Walkowiak et al. (2020), and number of amino acids (if full length) information for all copies identified within the 11 accessions.

Supplementary Table 2 Posterior probabilities and estimated omega (ω) values for all amino acid sites in gliadin alignments for each subgenome analyzed separately and together.

## References

[B1] Abrouk M. Athiyannan N. Müller T. Pailles Y. Stritt C. Roulin A. C. (2021). Population genomics and haplotype analysis in spelt and bread wheat identifies a gene regulating glume color. *Commun. Biol.* 4 1–11. 10.1038/s42003-021-01908-6 33742098 PMC7979816

[B2] Avni R. Nave M. Barad O. Baruch K. Twardziok S. O. Gundlach H. (2017). Wild emmer genome architecture and diversity elucidate wheat evolution and domestication. *Science* 357 93–97. 10.1126/science.aan0032 28684525

[B3] Blatter R. H. E. Jacomet S. Schlumbaum A. (2004). About the origin of European spelt (Triticum spelta L.): allelic differentiation of the HMW Glutenin B1-1 and A1-2 subunit genes. *Theor. Appl. Genet.* 108 360–367. 10.1007/s00122-003-1441-7 14564390

[B4] Branlard G. Dardevet M. Saccomano R. Lagoutte F. Gourdon J. (2001). Genetic diversity of wheat storage proteins and bread wheat quality. *Euphytica* 119 59–67. 10.1023/A:1017586220359

[B5] Brennan C. (2009). Gliadin and Glutenin: the unique balance of wheat quality. *Int. J. Food Sci. Technol.* 44 1865–1866. 10.1111/j.1365-2621.2007.01703.x

[B6] Call L. Kapeller M. Grausgruber H. Reiter E. Schoenlechner R. D’Amico S. (2020). Effects of species and breeding on wheat protein composition. *J. Cereal Sci.* 93:102974. 10.1016/j.jcs.2020.102974

[B7] Camerlengo F. Sestili F. Silvestri M. Colaprico G. Margiotta B. Ruggeri R. (2017). Production and molecular characterization of bread wheat lines with reduced amount of α-type gliadins. *BMC Plant Biol.* 17:248. 10.1186/s12870-017-1211-3 29258439 PMC5738072

[B8] Dubois B. Bertin P. Mingeot D. (2016). Molecular diversity of α-gliadin expressed genes in genetically contrasted spelt (Triticum aestivum ssp. spelta) accessions and comparison with bread wheat (T. aestivum ssp. aestivum) and related diploid Triticum and Aegilops species. *Mol. Breeding* 36:152. 10.1007/s11032-016-0569-5 27942245 PMC5104789

[B9] Dubois B. Bertin P. Muhovski Y. Escarnot E. Mingeot D. (2017). Development of TaqMan probes targeting the four major celiac disease epitopes found in α-gliadin sequences of spelt (Triticum aestivum ssp. spelta) and bread wheat (Triticum aestivum ssp. aestivum). *Plant Methods* 13:72. 10.1186/s13007-017-0222-2 28912827 PMC5588674

[B10] Dvorak J. Deal K. R. Luo M.-C. You F. M. von Borstel K. Dehghani H. (2012). The origin of spelt and free-threshing hexaploid wheat. *J. Hered.* 103 426–441. 10.1093/jhered/esr152 22378960

[B11] Escarnot E. Gofflot S. Sinnaeve G. Dubois B. Bertin P. Mingeot D. (2018). Reactivity of gluten proteins from spelt and bread wheat accessions towards A1 and G12 antibodies in the framework of celiac disease. *Food Chem.* 268 522–532. 10.1016/j.foodchem.2018.06.094 30064793

[B12] FAOSTAT (2021). Available online at: http://www.fao.org/faostat/en/#data/QC (accessed February 8, 2021).

[B13] Felsenstein J. (1985). Confidence limits on phylogenies: an approach using the bootstrap. *Evolution* 39 783–791. 10.2307/2408678 28561359

[B14] Geisslitz S. Longin C. F. H. Scherf K. A. Koehler P. (2019). Comparative study on gluten protein composition of ancient (einkorn, emmer and spelt) and modern wheat species (Durum and Common Wheat). *Foods* 8:409. 10.3390/foods8090409 31547385 PMC6769531

[B15] Gu Y. Q. Crossman C. Kong X. Luo M. You F. M. Coleman-Derr D. (2004). Genomic organization of the complex α-gliadin gene loci in wheat. *Theor. Appl. Genet.* 109 648–657. 10.1007/s00122-004-1672-2 15103408

[B16] Huo N. Zhu T. Altenbach S. Dong L. Wang Y. Mohr T. (2018). Dynamic evolution of α-Gliadin prolamin gene family in homeologous genomes of hexaploid wheat. *Sci. Rep.* 8:5181. 10.1038/s41598-018-23570-5 29581476 PMC5980091

[B17] International Wheat Genome Sequencing Consortium (IWGSC) (2014). A chromosome-based draft sequence of the hexaploid bread wheat (Triticum aestivum) genome. *Science* 345:1251788. 10.1126/science.1251788 25035500

[B18] International Wheat Genome Sequencing Consortium (IWGSC), Appels R. Eversole K. Stein N. Feuillet C. Keller B. (2018). Shifting the limits in wheat research and breeding using a fully annotated reference genome. *Science* 361:eaar7191. 10.1126/science.aar7191 30115783

[B19] Jordan K. W. Wang S. Lun Y. Gardiner L.-J. MacLachlan R. Hucl P. (2015). A haplotype map of allohexaploid wheat reveals distinct patterns of selection on homoeologous genomes. *Genome Biol.* 16:48. 10.1186/s13059-015-0606-4 25886949 PMC4389885

[B20] Juhász A. Belova T. Florides C. G. Maulis C. Fischer I. Gell G. (2018). Genome mapping of seed-borne allergens and immunoresponsive proteins in wheat. *Sci. Adv.* 4:eaar8602. 10.1126/sciadv.aar8602 30128352 PMC6097586

[B21] Kawaura K. Wu J. Matsumoto T. Kanamori H. Katagiri S. Ogihara Y. (2012). Genome change in wheat observed through the structure and expression of α/β-gliadin genes. *Funct. Integr. Genomics* 12 341–355. 10.1007/s10142-012-0269-0 22370744

[B22] Kim C.-Y. Quarsten H. Bergseng E. Khosla C. Sollid L. M. (2004). Structural basis for HLA-DQ2-mediated presentation of gluten epitopes in celiac disease. *Proc. Natl. Acad. Sci. U.S.A.* 101 4175–4179. 10.1073/pnas.0306885101 15020763 PMC384714

[B23] Kimura M. (1980). A simple method for estimating evolutionary rates of base substitutions through comparative studies of nucleotide sequences. *J. Mol. Evol.* 16 111–120. 10.1007/BF01731581 7463489

[B24] Kumar S. Stecher G. Li M. Knyaz C. Tamura K. (2018). MEGA X: molecular evolutionary genetics analysis across computing platforms. *Mol. Biol. Evol.* 35 1547–1549. 10.1093/molbev/msy096 29722887 PMC5967553

[B25] Li D. Jin H. Zhang K. Wang Z. Wang F. Zhao Y. (2018). Analysis of the Gli-D2 locus identifies a genetic target for simultaneously improving the breadmaking and health-related traits of common wheat. *Plant J.* 95 414–426. 10.1111/tpj.13956 29752764

[B26] Li H. Handsaker B. Wysoker A. Fennell T. Ruan J. Homer N. (2009). The sequence alignment/map format and SAMtools. *Bioinformatics* 25 2078–2079. 10.1093/bioinformatics/btp352 19505943 PMC2723002

[B27] Li Y. Xin R. Zhang D. Li S. (2014). Molecular characterization of α-gliadin genes from common wheat cultivar Zhengmai 004 and their role in quality and celiac disease. *Crop. J.* 2 10–21. 10.1016/j.cj.2013.11.003

[B28] Metakovsky E. Melnik V. Rodriguez-Quijano M. Upelniek V. Carrillo J. M. (2018). A catalog of gliadin alleles: polymorphism of 20th-century common wheat germplasm. *Crop. J.* 6 628–641. 10.1016/j.cj.2018.02.003

[B29] Noma S. Kawaura K. Hayakawa K. Abe C. Tsuge N. Ogihara Y. (2016). Comprehensive molecular characterization of the α/β-gliadin multigene family in hexaploid wheat. *Mol. Genet. Genomics* 291 65–77. 10.1007/s00438-015-1086-7 26159870

[B30] Ozuna C. V. Iehisa J. C. M. Giménez M. J. Alvarez J. B. Sousa C. Barro F. (2015). Diversification of the celiac disease α-gliadin complex in wheat: a 33-mer peptide with six overlapping epitopes, evolved following polyploidization. *Plant J.* 82 794–805. 10.1111/tpj.12851 25864460

[B31] Paape T. Briskine R. V. Halstead-Nussloch G. Lischer H. E. L. Shimizu-Inatsugi R. Hatakeyama M. (2018). Patterns of polymorphism and selection in the subgenomes of the allopolyploid Arabidopsis kamchatica. *Nat. Commun.* 9:3909. 10.1038/s41467-018-06108-1 30254374 PMC6156220

[B32] Paape T. Hatakeyama M. Shimizu-Inatsugi R. Cereghetti T. Onda Y. Kenta T. (2016). Conserved but attenuated parental gene expression in allopolyploids: constitutive zinc hyperaccumulation in the allotetraploid arabidopsis kamchatica. *Mol. Biol. Evol.* 33 2781–2800. 10.1093/molbev/msw141 27413047 PMC5062318

[B33] Payne P. I. (1987). Genetics of wheat storage proteins and the effect of allelic variation on bread-making quality. *Ann. Rev. Plant Physiol.* 38 141–153. 10.1146/annurev.pp.38.060187.001041

[B34] Petersen J. Kooy-Winkelaar Y. Loh K. L. Tran M. van Bergen J. Koning F. (2016). Diverse T Cell receptor gene usage in HLA-DQ8-Associated celiac disease converges into a consensus binding solution. *Structure* 24 1643–1657. 10.1016/j.str.2016.07.010 27568928

[B35] Petersen J. Montserrat V. Mujico J. R. Loh K. L. Beringer D. X. van Lummel M. (2014). T-cell receptor recognition of HLA-DQ2–gliadin complexes associated with celiac disease. *Nat. Struct. Mol. Biol.* 21 480–488. 10.1038/nsmb.2817 24777060

[B36] Pronin D. Börner A. Scherf K. A. (2021). Old and modern wheat (Triticum aestivum L.) cultivars and their potential to elicit celiac disease. *Food Chem.* 339:127952. 10.1016/j.foodchem.2020.127952 33152854

[B37] Qi P.-F. Chen Q. Ouellet T. Wang Z. Le C.-X. Wei Y.-M. (2013). The molecular diversity of α-gliadin genes in the tribe Triticeae. *Genetica* 141 303–310. 10.1007/s10709-013-9729-2 23892918

[B38] Quinlan A. R. Hall I. M. (2010). BEDTools: a flexible suite of utilities for comparing genomic features. *Bioinformatics* 26 841–842. 10.1093/bioinformatics/btq033 20110278 PMC2832824

[B39] Robinson J. T. Thorvaldsdóttir H. Wenger A. M. Zehir A. Mesirov J. P. (2017). Variant review with the integrative genomics viewer. *Cancer Res.* 77 e31–e34. 10.1158/0008-5472.CAN-17-0337 29092934 PMC5678989

[B40] Ronquist F. Teslenko M. van der Mark P. Ayres D. L. Darling A. Höhna S. (2012). MrBayes 3.2: efficient Bayesian phylogenetic inference and model choice across a large model space. *Syst. Biol.* 61 539–542. 10.1093/sysbio/sys029 22357727 PMC3329765

[B41] Ruiz-Carnicer A. Comino I. Segura V. Ozuna C. V. Moreno M. L. López-Casado M. A. (2019). Celiac immunogenic potential of α-Gliadin epitope variants from triticum and aegilops species. *Nutrients* 11:220. 10.3390/nu11020220 30678169 PMC6413208

[B42] Saitou N. Nei M. (1987). The neighbor-joining method: a new method for reconstructing phylogenetic trees. *Mol. Biol. Evol.* 4 406–425. 10.1093/oxfordjournals.molbev.a040454 3447015

[B43] Salentijn E. M. Goryunova S. V. Bas N. van der Meer I. M. van den Broeck H. C. Bastien T. (2009). Tetraploid and hexaploid wheat varieties reveal large differences in expression of alpha-gliadins from homoeologous Gli-2 loci. *BMC Genomics* 10:48. 10.1186/1471-2164-10-48 19171027 PMC2636828

[B44] Sánchez-León S. Gil-Humanes J. Ozuna C. V. Giménez M. J. Sousa C. Voytas D. F. (2018). Low-gluten, nontransgenic wheat engineered with CRISPR/Cas9. *Plant Biotechnol. J.* 16 902–910. 10.1111/pbi.12837 28921815 PMC5867031

[B45] Schaart J. G. Salentijn E. M. J. Goryunova S. V. Chidzanga C. Esselink D. G. Gosman N. (2021). Exploring the alpha-gliadin locus: the 33-mer peptide with six overlapping coeliac disease epitopes in Triticum aestivum is derived from a subgroup of Aegilops tauschii. *Plant J.* 106 86–94. 10.1111/tpj.15147 33369792 PMC8248119

[B46] Scherf K. A. Koehler P. Wieser H. (2016). Gluten and wheat sensitivities – An overview. *J. Cereal Sci.* 67 2–11. 10.1016/j.jcs.2015.07.008

[B47] Shan L. Molberg O. Parrot I. Hausch F. Filiz F. Gray G. M. (2002). Structural basis for gluten intolerance in celiac sprue. *Science* 297 2275–2279. 10.1126/science.1074129 12351792

[B48] Shewry P. R. (2018). Do ancient types of wheat have health benefits compared with modern bread wheat? *J. Cereal Sci.* 79 469–476. 10.1016/j.jcs.2017.11.010 29497244 PMC5824670

[B49] Shewry P. R. Halford N. G. (2002). Cereal seed storage proteins: structures, properties and role in grain utilization. *J. Exp. Bot.* 53 947–958. 10.1093/jexbot/53.370.947 11912237

[B50] Shiferaw B. Smale M. Braun H.-J. Duveiller E. Reynolds M. Muricho G. (2013). Crops that feed the world 10. Past successes and future challenges to the role played by wheat in global food security. *Food Sec.* 5 291–317. 10.1007/s12571-013-0263-y

[B51] Shimizu K. K. Copetti D. Okada M. Wicker T. Tameshige T. Hatakeyama M. (2020). De novo genome assembly of the Japanese wheat cultivar norin 61 highlights functional variation in flowering time and fusarium resistance genes in East Asian Genotypes. *Plant Cell Physiol*. 62 8–27. 10.1093/pcp/pcaa152 33244607 PMC7991897

[B52] Singh P. Arora A. Strand T. A. Leffler D. A. Catassi C. Green P. H. (2018). Global prevalence of celiac disease: systematic review and meta-analysis. *Clin. Gastroenterol. Hepatol.* 16 823.e2–836.e2. 10.1016/j.cgh.2017.06.037 29551598

[B53] Sollid L. M. Qiao S.-W. Anderson R. P. Gianfrani C. Koning F. (2012). Nomenclature and listing of celiac disease relevant gluten T-cell epitopes restricted by HLA-DQ molecules. *Immunogenetics* 64 455–460. 10.1007/s00251-012-0599-z 22322673 PMC3349865

[B54] Urade R. Sato N. Sugiyama M. (2018). Gliadins from wheat grain: an overview, from primary structure to nanostructures of aggregates. *Biophys. Rev.* 10 435–443. 10.1007/s12551-017-0367-2 29204878 PMC5899726

[B55] van Herpen T. W. Goryunova S. V. van der Schoot J. Mitreva M. Salentijn E. Vorst O. (2006). Alpha-gliadin genes from the A, B, and D genomes of wheat contain different sets of celiac disease epitopes. *BMC Genomics* 7:1. 10.1186/1471-2164-7-1 16403227 PMC1368968

[B56] Walkowiak S. Gao L. Monat C. Haberer G. Kassa M. T. Brinton J. (2020). Multiple wheat genomes reveal global variation in modern breeding. *Nature* 588 277–283. 10.1038/s41586-020-2961-x 33239791 PMC7759465

[B57] Yang Z. (2007). PAML 4: phylogenetic analysis by maximum likelihood. *Mol. Biol. Evol.* 24 1586–1591. 10.1093/molbev/msm088 17483113

[B58] Yang Z. Nielsen R. Goldman N. Pedersen A. M. (2000). Codon-substitution models for heterogeneous selection pressure at amino acid sites. *Genetics* 155 431–449. 10.1093/genetics/155.1.431 10790415 PMC1461088

[B59] Yang Z. Wong W. S. W. Nielsen R. (2005). Bayes empirical bayes inference of amino acid sites under positive selection. *Mol. Biol. Evol.* 22 1107–1118. 10.1093/molbev/msi097 15689528

