# Supplementary figures and images for: Multiple Wheat Genomes Reveal Novel Gli-2 Sublocus Location and Variation of Celiac Disease Epitopes in Duplicated α-Gliadin Genes
Source: Front Plant Sci. 2021 Sep 3;12:715985. doi: 10.3389/fpls.2021.715985 (PMC8446623; doi:10.3389/fpls.2021.715985)

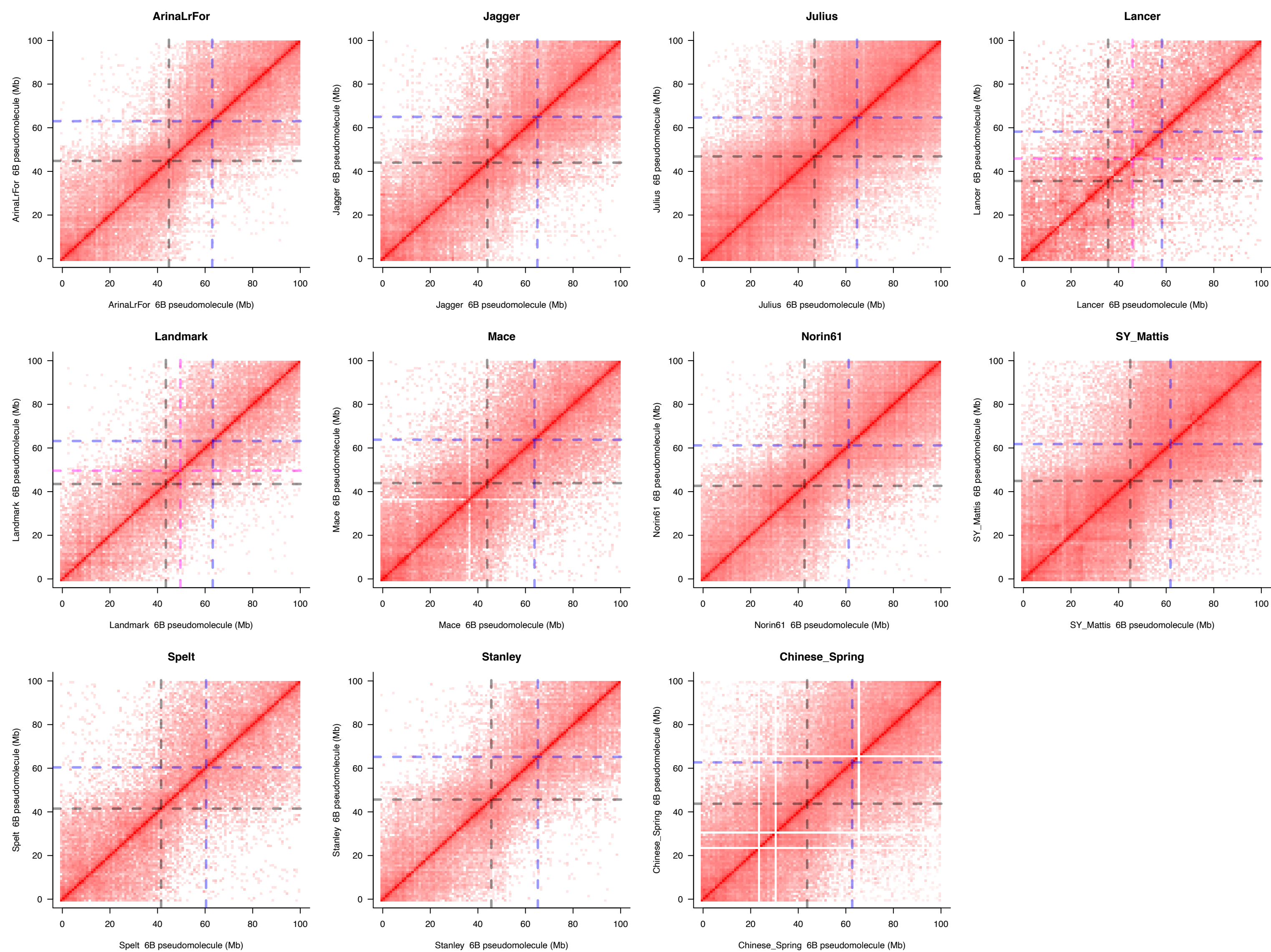

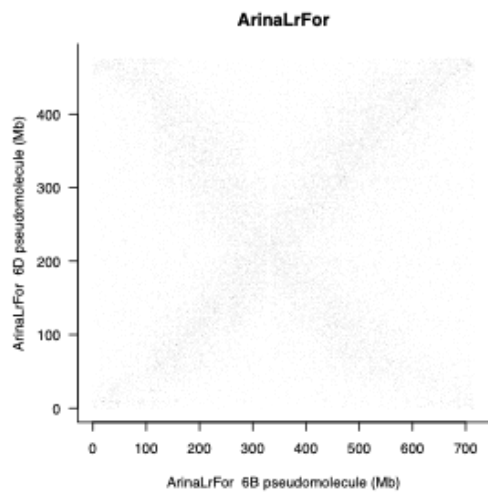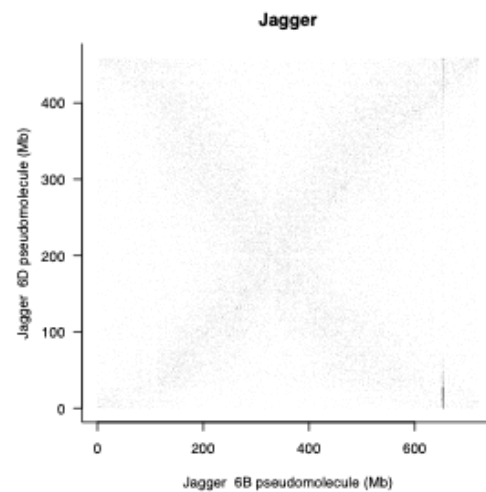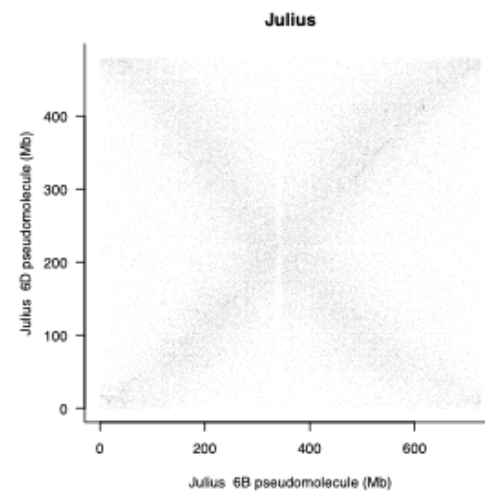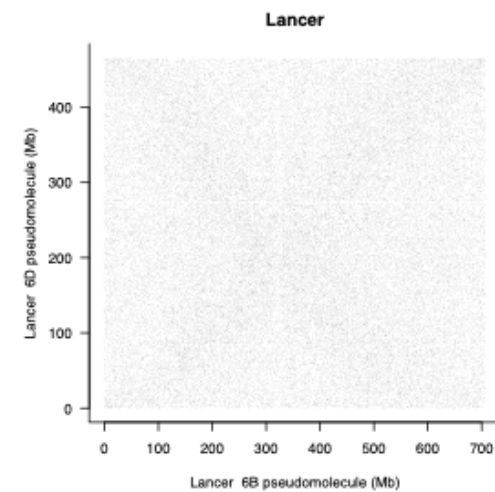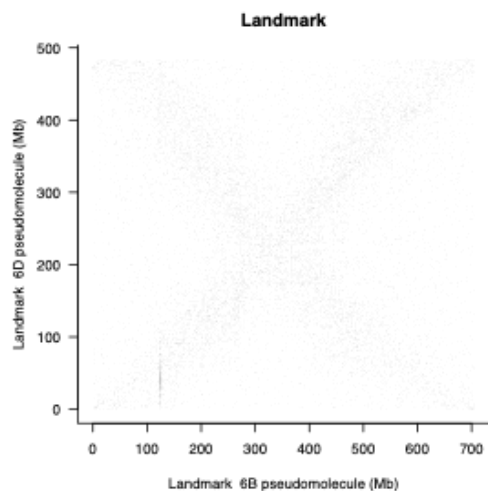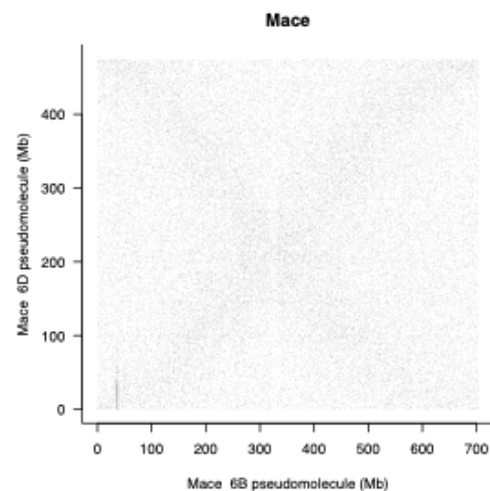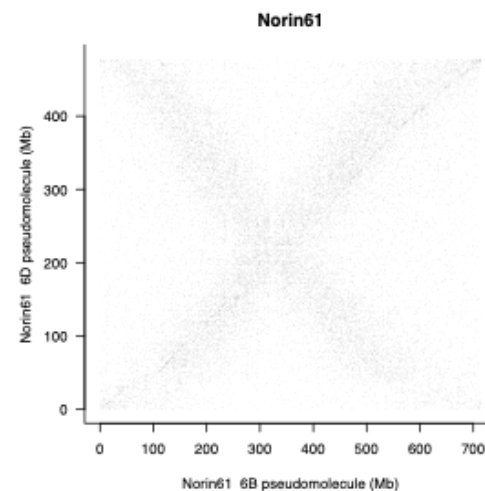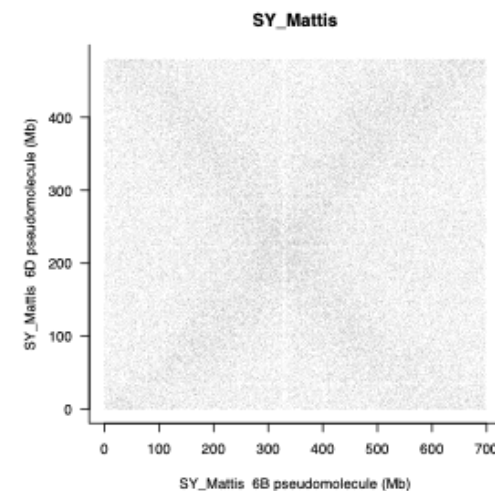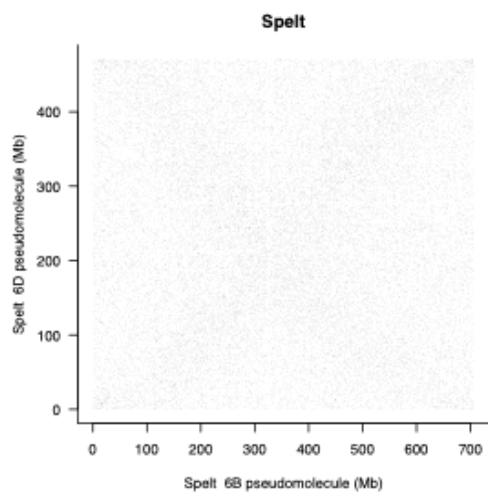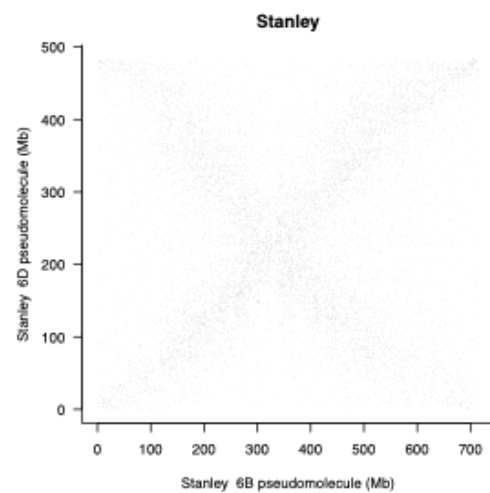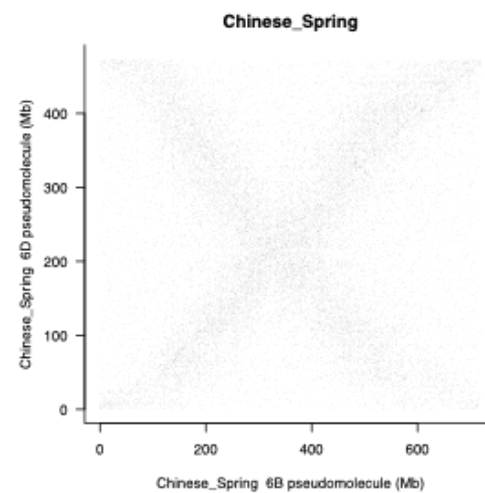

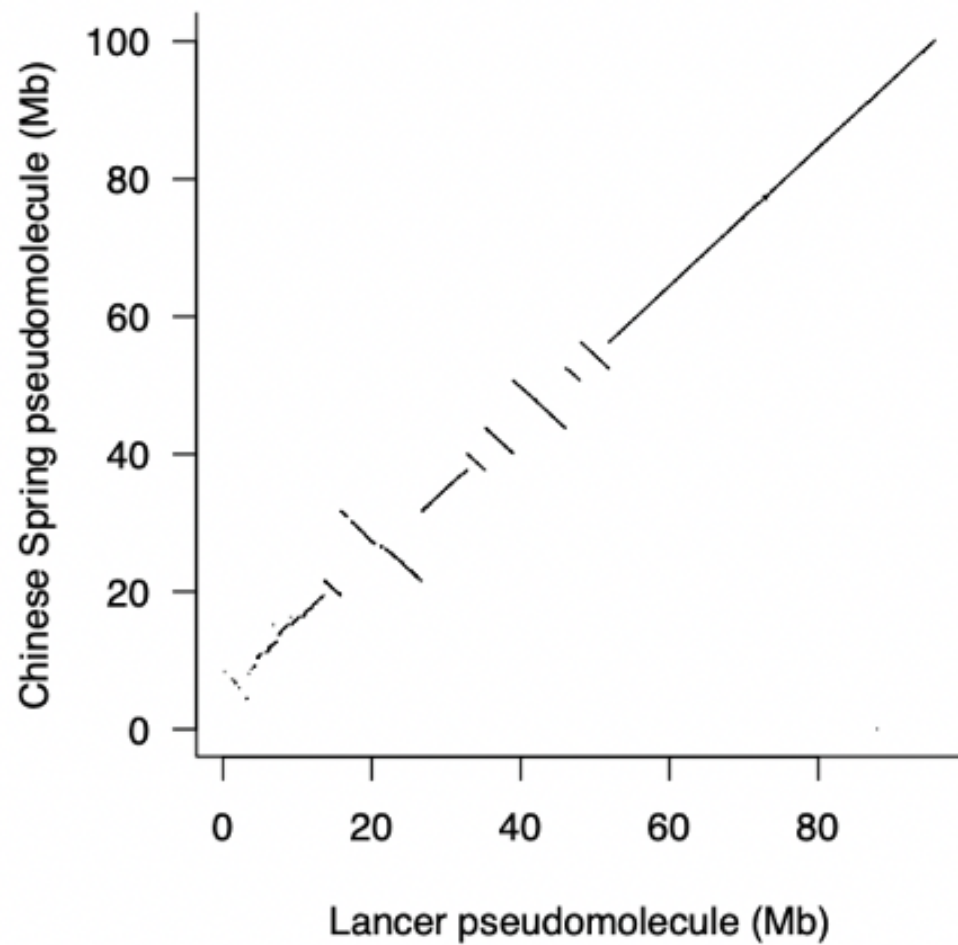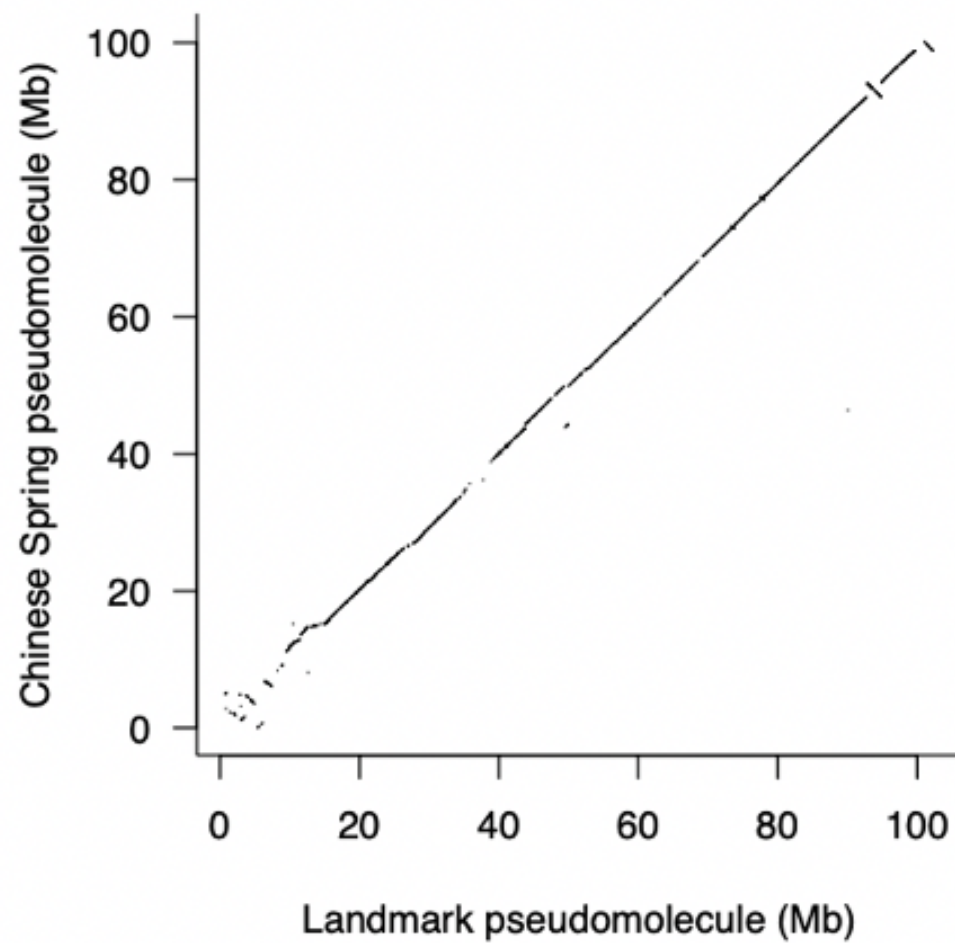

A

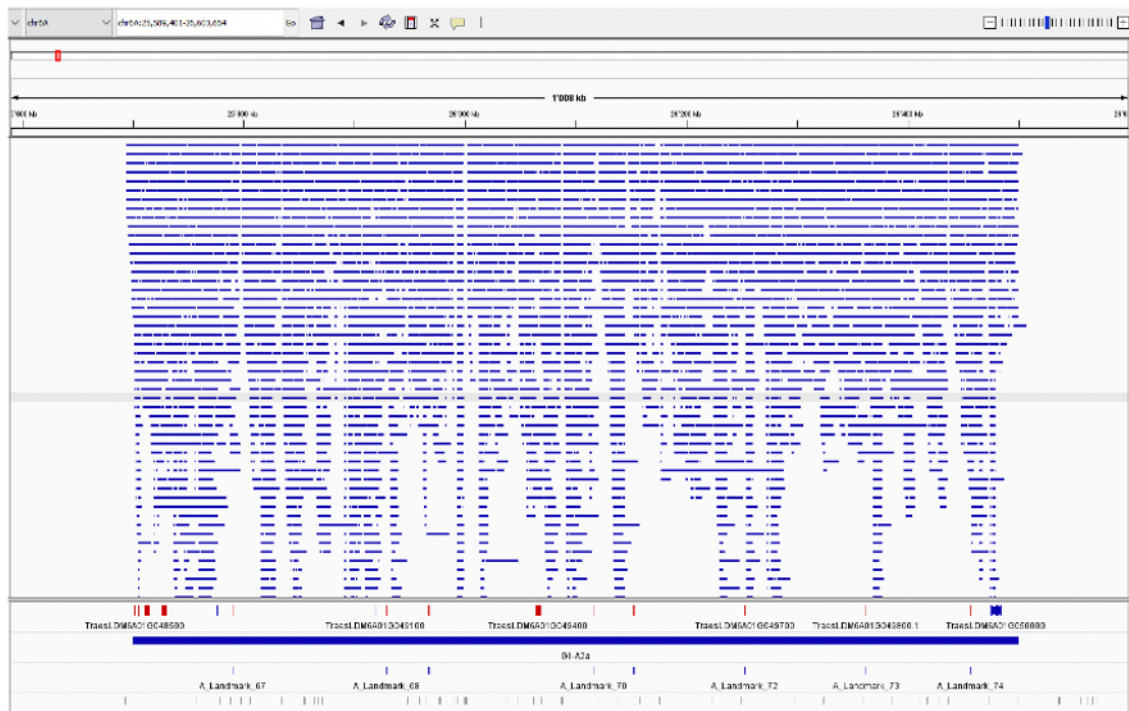

B

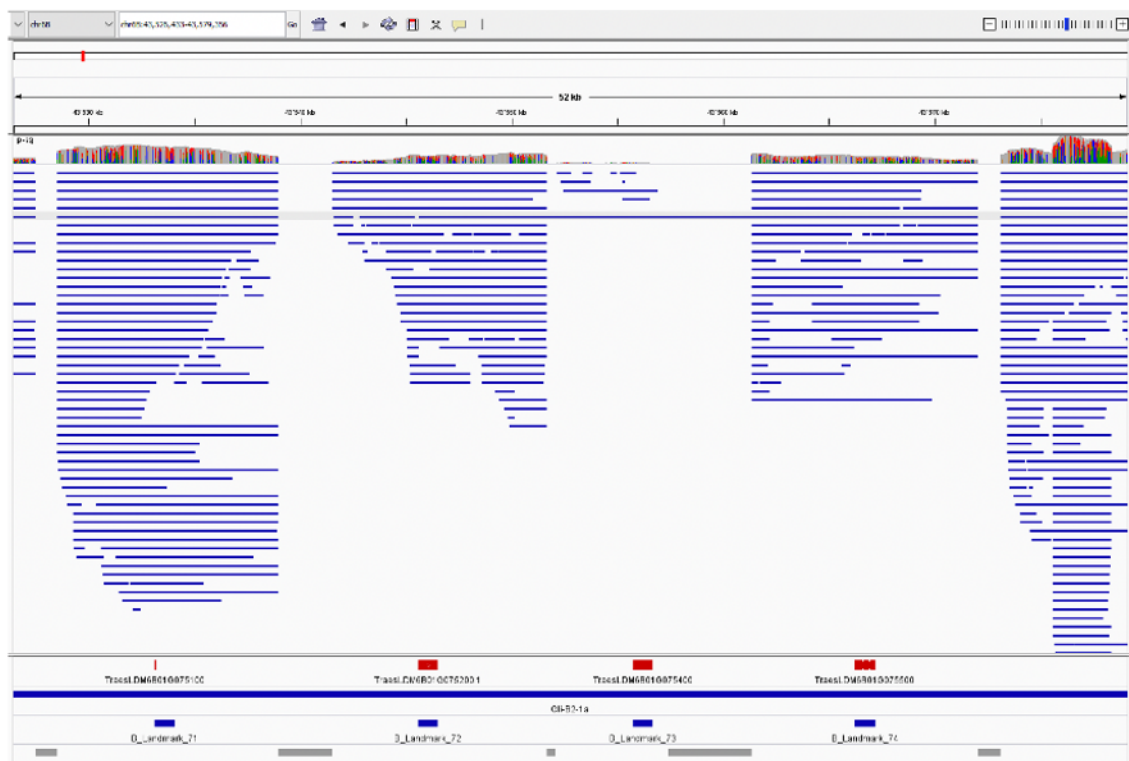

Supplement: Supplementary Figure 1 — Large-scale chromosome organization around the Gli-B2 loci in 11 accessions. Hi-C contact matrices of chromosome 6B at 0–100 Mb show a strong signal on the diagonal and a relatively even gradient perpendicular to the diagonal, indicating that the large-scale structure of assemblies is correct. Dashed lines represent the position of each sub locus identified in Gli-B2. [file Data_Sheet_1.PDF]
